# Patterns of Dysgraphia in Primary Progressive Aphasia Compared to Post-Stroke Aphasia

**DOI:** 10.3233/BEN-2012-110237

**Published:** 2013

**Authors:** Andreia V. Faria, Jenny Crinion, Kyrana Tsapkini, Melissa Newhart, Cameron Davis, Shannon Cooley, Susumu Mori, Argye E. Hillis

**Affiliations:** ^1^Department of RadiologyJohns Hopkins University School of MedicineBaltimoreMDUSA; ^2^Department of NeurologyJohns Hopkins University School of MedicineBaltimoreMDUSA; ^3^University College LondonLondonUK; ^4^Department of Physical Medicine and RehabilitationJohns Hopkins University School of MedicineBaltimoreMDUSA; ^5^Johns Hopkins UniversityBaltimoreMDUSA; ^6^Department of Cognitive ScienceJohns Hopkins UniversityBaltimoreMDUSA

**Keywords:** Dysgraphia, primary progressive aphasia, phonology, orthography, MRI

## Abstract

We report patterns of dysgraphia in participants with primary progressive aphasia that can be explained by assuming disruption of one or more cognitive processes or representations in the complex process of spelling. These patterns are compared to those described in participants with focal lesions (stroke). Using structural imaging techniques, we found that damage to the left extrasylvian regions, including the uncinate, inferior fronto-occipital fasciculus, and sagittal stratum (including geniculostriate pathway and inferior longitudinal fasciculus), as well as other deep white and grey matter structures, was significantly associated with impairments in access to orthographic word forms and semantics (with reliance on phonology-to-orthography to produce a plausible spelling in the spelling to dictation task). These results contribute not only to our understanding of the patterns of dysgraphia following acquired brain damage but also the neural substrates underlying spelling.

## 1. Introduction

Patterns of impairment in spelling have revealed distinct cognitive processes underlying normal spelling. For example, some patients after focal brain lesions are selectively unable to spell irregular words (e.g. spell leopard as lepperd), but are able to spell regular words and pseudowords (e.g. glimp). Others are able to spell both regular and irregular words, but cannot come up with a plausible spelling of an unfamiliar proper name or pseudoword. These patients provide evidence for relatively distinct mechanisms of sublexical phonology-to-orthography conversion and access to orthographic word forms (learned spellings of words). Other patients show modality-specific output impairments (impaired written spelling with intact oral spelling), indicating that the problem is in accessing a letter shape or motor plan. Still others have a very generalized impairment in oral and written spelling of words and pseudowords that is dependent on the number of letters in the stimulus, which can be explained by an impairment in working memory or a "buffer" system for holding the sequence of abstract letter identities while the word is written or spelled aloud.

Most of these patterns have been described in chronic stroke patients with large lesions. It has been difficult to localize the lesions associated with distinct components of the spelling process, although Rapcsak and colleagues have found that chronic left perisylvian lesions (supramarginal gyrus, superior temporal gyrus, posterior inferior frontal gyrus, and precentral gyrus) are associated with impaired phonology-to-orthography; while extra-striate lesions are associated with impairments in other components that result in reliance on phonology-to-orthography (and "regular" or sublexical spelling) [1]. Some fMRI studies of spelling

ISSN 0953-4180/13/$27.50 © 2013 – IOS Press and the authors. All rights reserved have supported these hypotheses [2]. In a large study of acute stroke, we did not find distinct regions associated with pseudoword and word spelling; however, few participants in that study showed a dissociation between word and pseudoword spelling, so there was insufficient power to detect distinct regions associated with one or the other [3]. In a separate series, we did find that lesions of posterior inferior frontal cortex were associated with impaired sublexical phonology-to-orthography conversion in some participants [4,5].

In this paper we report patterns of dysgraphia very similar to the patterns that have been reported in chronic unilateral stroke, but in participants with primary progressive aphasia (PPA), a neurodegenerative disease that disproportionately affects language for at least two years before the onset of other cognitive symptoms [6]. These data are of interest, because they demonstrate that these patterns of spelling impairment observed in both patient populations cannot be accounted for by rehabilitation alone. As PPA affects somewhat distinct areas of brain that are not typically affected by vascular lesions, they also provide the opportunity to look for unique patterns, not seen after unilateral focal stroke. Using structural imaging techniques, we found that PPA participants who relied on phonology-to-orthography conversion for successful spelling could be distinguished from those who were disproportionately impaired in phonology-to-orthography conversion. Damage to the left extrasylvian regions was significantly associated with impairments in access to orthographic word forms and semantics (with reliance on phonology-to-orthography to produce a plausible spelling in the spelling to dictation task) including the uncinate, inferior fronto-occipital fasciculus, and sagittal stratum (including geniculostriate pathway and inferior longitudinal fasciculus), as well as other deep white and grey matter structures. These imaging data directly complement the imaging obtained from chronic stroke participants and furthers our understanding of the role of the left perisylvian regions involved in spelling.

## 2. Methods

### 2.1. Participants

We enrolled a series of 30 participants with PPA who were seen in the senior author's outpatient clinic and agreed to participate. They were diagnosed with PPA on the basis of having a predominant and progressive deterioration in language in the absence of major change in personality, behavior or cognition other than praxis for at least two years [6]. Participants were classified, when possible, as one of the variants of PPA according to recent guidelines [7]. However, several participants were only anomic and dysgraphic, and did not meet criteria for any of these variants. PPA variants and demographics are included in Table 1. All participants completed a battery of language and spelling tests together and had a high resolution T1 weighted image (T1-WI) brain scan, within 1 month post testing. Six participants were followed up one year later where they repeated the scan and testing.

Table 1
PPA variants and demographic information

| Patient ID | Age (years) | Gender | PPA variants | Locus of impairment |
|---|---|---|---|---|
| 1 | 62 | Male | sv | 1 |
| 2 | 56 | Female | sv | 1 |
| 3 | 68 | Male | sv | 1 |
| 4 | 84 | Female | nfv | 2 |
| 5 | 62 | Female | lv | 3 |
| 6 | 70 | Male | nfv | 4 |
| 7 | 58 | Male | lv | 3 |
| 8 | 67 | Female | lv | 3 |
| 9 | 74 | Female | nfv | 1 |
| 10 | 66 | Female | lv | 3 |
| 11 | 60 | Female | sv | 1 |
| 12 | 84 | Female | nfv | 3 |
| 13 | 70 | Male | lv | 3 |
| 14 | 62 | Male | sv | 1 |
| 15 | 74 | Female | sv | 1 |
| 16 | 67 | Female | lv | 3 |
| 17 | 71 | Male | lv | 3 |
| 18 | 70 | Male | sv | 1 |
| 19 | 72 | Female | lv | 3 |
| 20 | 71 | Female | lv | 1 |
| 21 | 70 | Female | unclassifiable | 1 |
| 22 | 73 | Female | lv | 1 |
| 23 | 69 | Female | unclassifiable | 1 |
| 24 | 51 | Male | lv | 1 |
| 25 | 79 | Female | unclassifiable | 3 |
| 26 | 62 | Female | nfv | 3 |
| 27 | 57 | Male | sv | 1 |
| 28 | 55 | Female | nfv | 4 |
| 29 | 63 | Female | lv | 4 |
| 30 | 73 | Male | sv | 1 |

PPA variants: sv = semantic; nfv = nonfluent/agrammatic; lv = logopenic.
Locus of impairment: 1 = semantics & orthography; 2 = Phonology to Orthography Conversion (POC); 3 = partially impaired POC, partially impaired access to orthographic word forms; 4 = impairment at the level of the Graphemic Buffer.

### 2.2. Language testing

A battery of tests was used to classify participants' overall language impairments and aid classification into the global PPA variant groupings, as described in Gorno-Tempini et al. [7]. These included 1) *speech production*: analysis of the Cinderella story, elicited with a series of pictures; naming of nouns and verbs; repetition of sentences; a sentence formulation test using scrambled words or anagrams of sentences; 2) *reading*: oral reading; reading aloud of regular and irregular words; 3) *language comprehension*: a short form of the Pyramids and Palm Trees Test [19], and a spoken word to picture matching test. For detailed analyses of spelling the Johns Hopkins Dysgraphia Battery was administered to all participants (see [8] for details of the stimuli). Within each subtest of this battery stimuli control for a multitude of variables including word frequency, length and grammatical class. For example, in the word length subtest, 4, 5, 6, 7, and 8-letter words are matched for frequency, length in syllables, and grammatical word class.

*Spelling error classifications*: Errors were classified as phonologically plausible errors (PPEs) if each letter produced by the participant was a valid instance of the phoneme (speech sound) in the corresponding stimulus at that position. For example, for the stimulus "cactus" phonologically plausible errors included kaktis, kactos, caktess, cacktuss, etc. Phonologically implausible nonword errors were nonwords in which at least one letter did not correspond to the phonology of the stimulus (e.g. "cactus" → kaptess). Semantic errors were semantically related to the word (e.g. "cactus" → plant or desert). Phonologically similar word errors (e.g. bear → pear) shared at least 50% of phonemes, and excluded morphologically related errors (e.g. read → "reading"). Morphological errors included derivational errors (e.g. baking → baker) and inflectional errors (e.g., baking → baked, the latter of which did not change the grammatical word class).

*Determining the locus of impairment within the spelling system*: Our *a priori* framework for identifying the level of impairment within the spelling system included the types of errors in each spelling task that were expected and the pattern of errors across tasks. Nevertheless, the precise criteria are somewhat difficult to define because the errors also depend in part on the severity as well as the locus of impairment. Even when criteria can be defined, often a patient may not meet every single criterion and yet will overall have the impression of having a pattern of performance that can be nicely explained by assuming a deficit to a particular component of the spelling process. So, we refer readers to criteria for each deficit outlined in Beeson and Hillis [8], but these will be best illustrated in each case below.

We identified lexical effects or word length effects in spelling using Fisher's exact tests, comparing correct versus incorrect responses on lists contrasting the relevant contrast, matched for the other variables that might affect performance. The patient's first response was scored; there were no time limits for responding.

### 2.3. Imaging analysis

MPRAGE T1-WIs (TR/TE = 8.4/3.9 ms) were acquired using a 3T whole body MRI scanner (Philips Medical Systems, Best, The Netherlands), with axial orientation and a image matrix of 256 × 256 mm. Half of the participants were scanned with a field of view (FOV) of 230 × 230 mm and 120 slices of 1 mm thickness; and half had FOV of 212 × 212 mm and 140 slices of 1.1 mm thickness. To measure the volume of each anatomical region we performed an atlas-based analysis (ABA). In brief, the ABA consists on transforming a brain image (the atlas) and the anatomical parcellation defined in this atlas to each participant's brain. As a result, a specific parcellation is created for each individual and, therefore, each participant's brain can be fully and automatically parceled in multiple regions of interest (ROIs). In this study, the ABA analysis was made possible due the high accuracy of the mapping algorithm, the large deformation diffeomorphic metric mapping – LDDMM [13]. As we showed in previous studies [9–11], the accuracy of this automated parcellation rivals the manual delineation of structures, considered as gold standard.

A schematic diagram of the imaging post-process, performed using DiffeoMap (Li, X.; Jiang, H.; and Mori, S.; Johns Hopkins University, www.MriStudio.org or mri.kennedykrieger.org), is shown in Fig. 1. The images were first normalized to the ICBM-DTI-81 coordinates [12] using a 12-parameter affine transformation and further transformed non-linearly to a single-subject template using LDDMM. The dual-contrast LDDMM [13] was based on T1-WIs and cerebrospinal fluid (CSF) maps. The JHU-MNI "Eve" was chosen as the Atlas. This is a single-subject template in the ICBM-DTI-81 space, extensively parceled and labeled to 159 regions. Details of this parcellation are described in our previous article [14]. Because of the reciprocal nature of the LDDMM, the transformation results can be used to warp the parcellation map to the original MRI data, thus automatically parceling each brain into the 159 sub-regions. After sub segmenting the cortex and the associated white matter in peripheral ROIs using

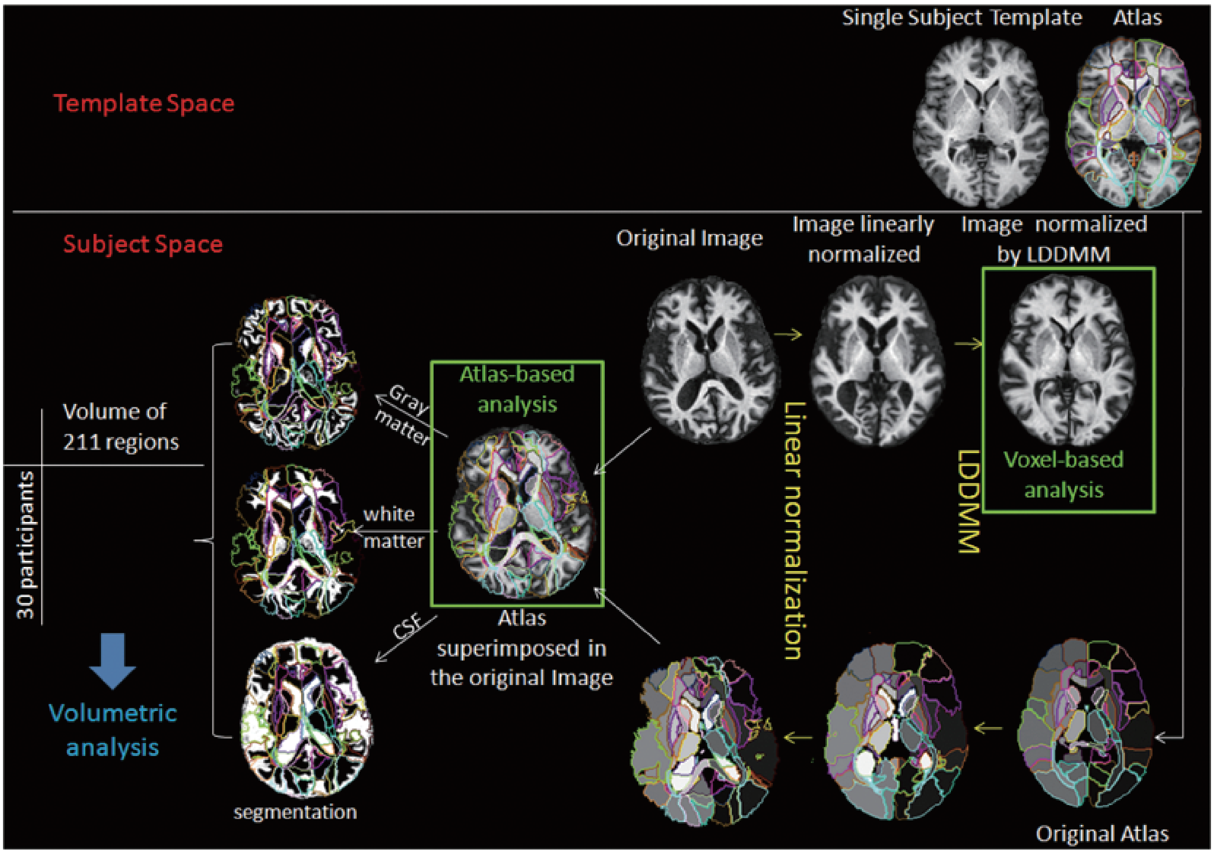

Fig. 1. Image post-processing. Using linear transformations and large deformation diffeomorphic metric mapping (LDDMM), we mapped each participant's brain to the atlas space. After this procedure, all brain images were transformed to a shape similar to that of the atlas, where is possible to perform voxel-based analysis. Using the reciprocal attribute of LDDMM, the parcellation map was transformed to each original MRI. This allows automated segmentation of the original images into 159 subregions. For cortical areas where there is a large amount of anatomical variability, the cortex and the white matter were further divided in each participant using SPM8 segmentation. This resulted in a matrix of participants by volume of 211 regions that was used for the volumetric analysis.

tissue maps obtained from SPM8, we finally obtained 3 dimensional 211 ROIs for each participant.

The post-processing of the six participants for whom longitudinal imaging data were collected followed the same procedure except in these cases we did not use "Eve" as the target but each participant's first T1-WI. Here we were interested in mapping volumetric within-subject changes over time and relating this to their corresponding changes in spelling. As a quantitative metric of local volume changes, we used the Jacobian determinant (i.e., the local expansion factor) of the LDDMM deformation fields. The Jacobian maps indicate local tissue expansion (Jacobian $> 1$) or shrinkage (Jacobian $< 1$) relative to the template [15,16] that allows identification of localized volume increases/reductions at the voxel level.

The native differences in the ROIs volumes were evaluated using ANOVA. Age, gender and image protocol were co-variates of no interest. In a second set of analyses, the ROIs volumes were normalized by the total brain volume to exclude any confounding effects of total intracranial volume. The level of statistical significance was set at $p$-value $< 0.01$ throughout unless otherwise reported.

## 3. Results

First we report three distinct patterns of spelling impairments among the PPA participants that can be explained by proposing disruption at the level of one or two cognitive representations or processes within the complex spelling system. Then we report the group's structural imaging results for participants who made predominantly phonologically plausible errors (i.e. rely on phonology to orthography conversion) versus those who made predominantly implausible errors (impaired phonology to orthography conversion as well as partially impaired access to orthographic word forms).

### 3.1. Pattern 1: Impairment at the level of orthography and semantics

When either access to semantics or access to orthographic word forms (or both) is impaired, we would expect reliance on sublexical phonology-to-orthography conversion mechanisms. Of course, there might be an interaction between lexical and sublexical mechanisms, such that participants use a combination of information to access lexical representations when they have partial impairment of one or the other mechanism [17, 18]; we address that issue in more detail in the subsequent sections. Here we describe three PPA participants who seemed to rely almost entirely on sublexical mechanisms to spell. It is important to highlight that orthography to phonology conversion (OPC) was spared, because participants were able to spell pseudowords.

*Case 1* is a 62 year old man with 20 years of education who was first evaluated about four years after the onset of anomia. He had noticed deterioration in word retrieval and spelling but was still working in a highly verbal job. At that time, he showed preservation of word and sentence comprehension, sentence repetition, and conceptual semantics. He lived independently and remained successfully employed in a high level of occupation.

His performance on the Johns Hopkins Dysgraphia Battery could be explained by selective impairment at the level of accessing orthographic word forms, with reliance on sublexical phonology-to-orthography conversion mechanisms to spell. Nearly all (92.3%) of his errors were phonologically plausible (e.g. sparrow → sparo; courage → currage; palace → pallis; bought → bot; about → abowt; become-> becum; career → currear). Furthermore, he spelled pseudowords more accurately than words (91.2% vs. 63.1; $p < 0.005$); regular words more accurately than irregular words (93.3 vs. 63.8%; $p < 0.005$). At that time, there was no difference in spelling accuracy for concrete vs. abstract words (52.4 vs. 57.1%) or any effect of word length (e.g. 4-letter vs. 8-letter words: 64.3 vs. 42.9%).

One year later, Case 1 showed deterioration in his word and sentence comprehension. He scored only 3/17 correct on a word/picture verification test, on which virtually all non-neurologically impaired adults make no errors. He correctly accepted most targets as the name of the object, but also incorrectly accepted nearly all semantically related words (e.g. "knife" for fork) as the name of the object. On a 15 item version of the Pyramid and Palm Trees Test [19], a picture association test, he scored on 12/15 (normal performance is ceiling). He continued to live independently, but made errors in daily life (e.g., offered his credit card when asked for his insurance card). His speech remained fluent, grammatical, and well articulated. He was able to carry on a social conversation, but produced few specific nouns. He made frequent semantic paraphasic errors and circumlocutions when speaking. He named verbs (40%) more accurately than nouns (13%). At this time point he met the criteria for semantic variant PPA (svPPA) and his spelling had deteriorated substantially as well. He still spelled pseudowords more accurately than words (79.4% vs. 57.1%; $p = 0.03$); and there was no effect of concreteness, grammatical word class, or frequency. All responses showed some attempt to apply phonology to orthography conversion mechanisms. Figure 2 shows deterioration of his accuracy on subtests of the Dysgraphia Battery over the course of one year. Related to his dramatic deterioration in semantics, the imaging data showed significant areas of brain atrophy between the two testing sessions, primarily in the left frontal and temporal regions.

*Case 2* is a 56 year old right handed woman with a master's degree who was in graduate school when she developed progressive difficulty with word retrieval and understanding what she was reading. She remained independent in daily activities for several years, but needed to discontinue her studies. She was eventually diagnosed with svPPA on the basis of her impaired word comprehension with spared speech articulation and speech fluency. She was scanned and tested twice on the dysgraphia battery, one year apart, although not all lists were given the second time due to her significant deterioration over that time. Her speech was fluent and grammatical. She remained very pleasant and cooperative, although it was difficult for her to understand words and the directions for new tasks. Her husband needed to take over the cooking, driving, and housework, because she also developed difficulties understanding the meanings of objects.

On both testing occasions, the majority of her spelling errors were phonologically plausible. PPE's constituted 82.6% of errors at time 1 and 80.0% of her errors at time 2. Errors included: ocean → ousean, strange → strainge, column → callem, sought → saut, thief → theaf, jerk → jurck. Orthographic probability did not significantly affect accuracy, although she was more accurate for high probability than low probably

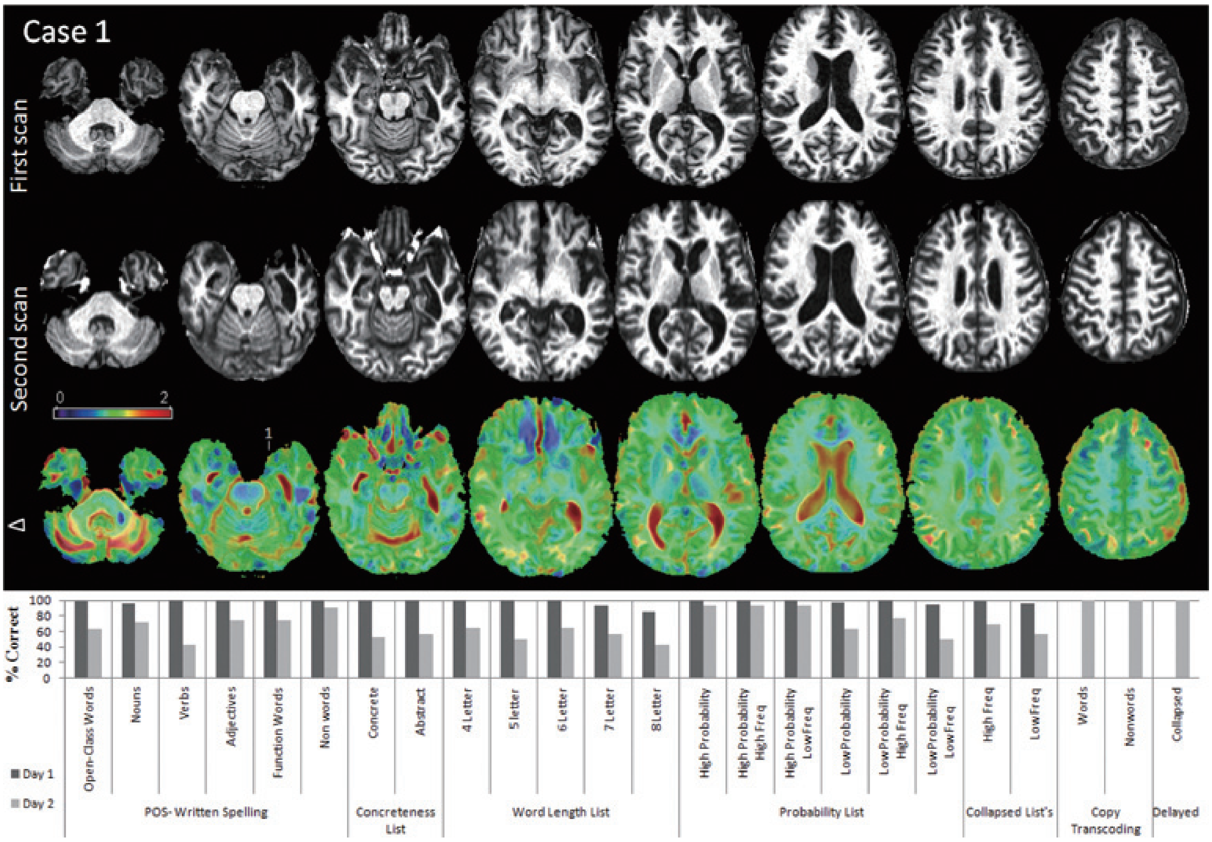

Fig. 2. Longitudinal volumetric analysis of Case 1. Note the enlargement of lateral ventricles, particularly at left, between the first and the second scans, spaced by 1 year. $\Delta$ is the color-coded for the Jacobians determinants from LDDMM and shows how each voxel expanded (red, $> 1$) or shrieked (blue, $< 1$) overtime. Note the "expansion" of the ventricles, in agreement with the visual impression, and also the atrophy (blue patches) in frontal and temporal areas. The bottom row shows the results of spelling on various subtests of the Dysgraphia Battery Test, 1 year apart.

words: 83.3% vs. 71.2% at time point 1; this list was not administered at time 2. The only variables to significantly affect accuracy were lexicality and concreteness. She spelled pseudowords significantly more accurately than words (97.1% vs. 76.2%, $p = 0.007$ at time 1; 73.6% vs. 42.9%, $p = 0.004$ at time 2) and abstract words more accurately than concrete words (81.0 vs. 71.4% at time 1; 76.2% vs. 33.3%, $p = 0.01$) at time 2. Figure 3 shows deterioration of her accuracy on subtests of the Dysgraphia Battery and the progression of brain atrophy temporal, parietal (particularly left) and subregions of basal ganglia over the course of 1 year.

*Case 3* is a 68-year-old man with a college education who was tested 8 years after onset of aphasia, when he met criteria for svPPA. He had fluent, grammatical, but "empty" speech with little content and only a shallow awareness of his difficulty communicating. He had impaired word and sentence comprehension. He lived with his wife, but was independent in daily activities, including driving. His performance fell somewhere between the first and second pattern shown by Case 1. Only 38% of his errors were phonologically plausible errors (e.g., center → senter; ready → readie), but many others were "close" (e.g., complete → compleade; fluid → floude; future → fercher; frequent → freacken). He spelled pseudowords more accurately than words (38.2% vs. 9.5% correct; $p < 0.001$). However, there were no significant effects of any tested parameters for words, because his performance was essentially at floor. For example, there was no difference between regular and irregular words because both were very poorly spelled (10% correct for each). Abstract words were spelled slightly more accurately than concrete words (14% vs. 0 correct), but there was no effect of word length (0% correct for both 4- and 8-letter words). His profile suggested an attempt to rely on phonology to orthography conversion mechanisms to spell.

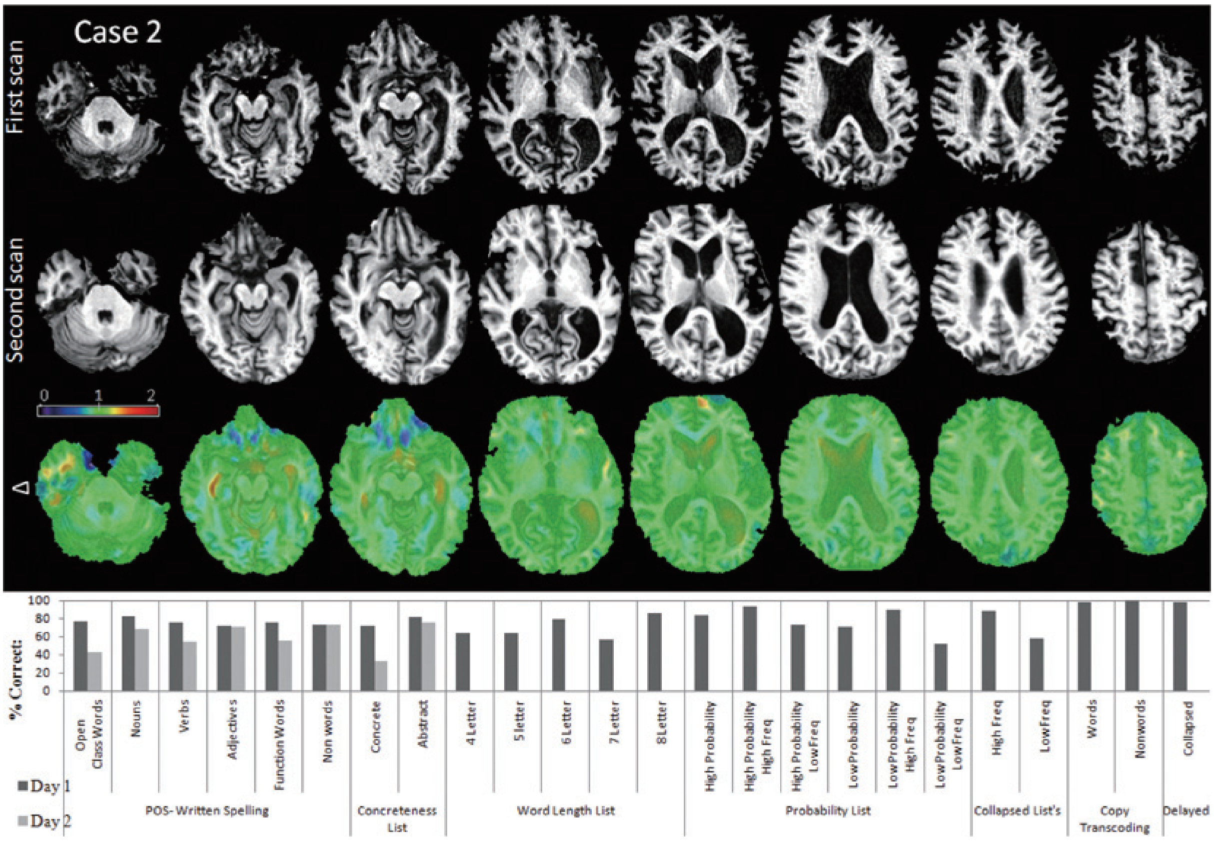

Fig. 3. Longitudinal volumetric analysis of Case 2. Again, first and second scans are spaced by 1 year and Δ shows how each voxel changed overtime (red (> 1) = expansion; blue (< 1) = shrinkage). Note the enlargement of CSF spaces (ventricles and sulci) and the atrophy of temporal, parietal (particularly left) and subregions of basal ganglia. The bottom row shows the results of spelling on various subtests of the Dysgraphia Battery Test, 1 year apart.

In summary, these 3 PPA cases' spelling profiles are similar and consistent with those of chronic stroke participants previously reported in the literature. For example, patient JJ, reported by Hillis and Caramazza [17], had a category-specific semantic impairment, affecting naming and comprehension of all categories except animals (and to a lesser degree, also sparing fruits and vegetables). He had no special premorbid expertise, familiarity, or fondness for animals according to his wife; he had no pets and had not visited a zoo in his adult life. Nevertheless, he was remarkably accurate in naming and word/picture matching for all types of animals (77–100%) compared to inanimate objects (8–33% correct), but he spelled to dictation non-animal names correctly (for regular names) or plausibly (e.g. 'carrot' → *cairit,* 'kangaroo' → *cangarue*). He made the same sorts of errors in written naming (e.g. ostrich → ostrage), although he also made semantic errors (e.g. bean → pea) or mixed semantic + PPE (e.g. carrot → cyoucumber) in written naming for non-animals. Virtually all of his errors in spelling to dictation (and reading) were PPEs. He occasionally spelled or read-aloud irregular words that he only partially understood. This was interpreted as evidence that access to lexical representations for output can be achieved through a summation of partial information from the semantic system and partial information from phonology-to-orthography and orthography-to-phonology conversion mechanisms. JJ's stroke affected a large part of the left anterior and inferior, middle, and superior temporal cortex, and basal ganglia, see [17].

### 3.2. *Pattern 2: Severe impairment at the level of phonology to orthography conversion* (*and partially impaired access to orthographic word forms for output*)

*Case 4* is an 84 year old woman 9 years post onset of nonfluent/agrammatic variant PPA (nfvPPA), who had a master's degree in a health care related field. She lived independently in a retirement community. She had effortful, poorly articulated, telegraphic speech, and named objects more accurately than actions. Sentence production was agrammatic. She had asyntactic comprehension, but followed conversation well. On the Dysgraphia Battery, she showed nearly the opposite pattern to the previously described participants. She spelled words more accurately than pseudowords (51.2 vs. 0%; $p < 0.05$). Only 1.6% of her errors could be considered PPEs. Her most common error type (43.2%) was semantic (e.g. beauty → pretty; happy → laughter; tiny → little; jury → plea; college → graduation; bought → store; moose → deer; debt → money). Her other errors consisted of phonologically implausible words (10.3%), phonologically similar words (21.1%; e.g. belief → between; should → short; offense → often; palace → place), or "don't know" (23.8%). There was a non-significant trend toward concrete words to be spelled more accurately than abstract words (76.2 vs. 47.6%), again the opposite pattern to that seen in the participants described above who had svPPA. There was no effect of word length (e.g. 4-letter vs. 8-letter: 50.0 vs. 57.1%). The only variable to significantly affect spelling accuracy was grammatical word class: she spelled nouns significantly more accurately than verbs at both time point 1 (67.9% vs. 35.7% correct; $p = 0.02$) and again 1 year later (25.0% vs. 3.6% correct; $p = 0.02$).

This identical pattern has been reported in an acute stroke patient RCM [5]. RCM had very poor phonology to orthography conversion (POC) and made semantic errors in spelling to dictation and written, despite intact word comprehension and oral naming of the same items. She also showed an effect of concreteness, spelled nouns better than verbs, and showed no effects of other variables. RCM had a lesion in the left posterior inferior frontal cortex (Broca's area). This pattern of performance, with both impaired access to POC mechanism and written word forms (especially verbs) for output was also described in an additional group of stroke patients with acute ischemia in Broca's area [20]. In these patients, impaired spelling of words and pseudowords was associated with infarct and/or hypoperfusion in voxels within Brodmann's areas 44/45 (Broca's area), the area of cortical atrophy classically associated with nfvPPA, see [7]. In a separate large acute stroke study [3], damage to the left supramarginal gyrus, Brodmann's area 40, was also associated with this spelling profile.

### 3.3. *Pattern 3: Partially impaired access to orthographic word forms and partially impaired phonology to orthography conversion mechanisms*

In some cases, there is nearly equal impairment of lexical and sublexical mechanisms. Participants make some phonologically plausible errors (PPEs) and at least as many implausible errors. They make phonologically related word errors also, probably because partial information from OPC mechanisms can be used to access both target words and phonologically related words in the lexicon. Because they have intact semantics, their intact semantic features also activate both the target word and semantically related words in the lexicon, so that semantically related words may be activated. Words that are both semantically and phonologically related to the target are common errors, resulting from an interaction from partial information from (impaired) POC mechanisms and an intact lexical-semantic system, activating representations whose access is impaired.

To illustrate, *Case 5* is a 62 year old woman 6 years since onset of logopenic variant PPA (lvPPA), characterized by fluent, grammatical speech, with intact word comprehension but poor sentence repetition. She lived with her husband, but was independent in daily activities. She was very pleasant and interactive, and initially had good recall of events. She made frequent circumlocution and phonemic paraphasic errors and hesitations when speaking.

On the dysgraphia battery, she spelled words and pseudowords with approximately equal accuracy (65.5 vs. 61.8% accuracy). There was no significant difference between high and low regularity words (80.0 vs. 83.8% correct). There was no significant effect of word length; she correctly spelled 85.7% of 4-letter vs. 92.9% of 8-letter words.

Only 15.2% of her errors were phonologically plausible errors (e.g. rooster → rouster; pigeon → picheon). The majority of her errors were phonologically similar word errors (e.g. bright → bride, brick; chain → chant) or phonologically/semantically similar words including morphological errors (e.g. jury → juror, absence → absent; speak → speech; begin → begun). These types of errors have been proposed to occur as a result of an interaction between partial information from the semantic system and partial information from the impaired orthography-to-phonology conversion mechanisms, while accessing lexical representations semantically or phonologically related to the target. There-

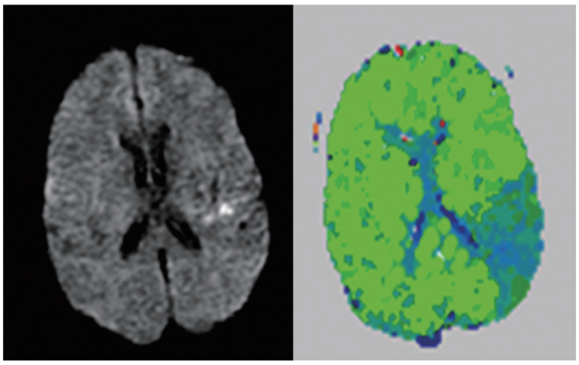

Fig. 4. Diffusion-weighted image (left) and Perfusion-weighted image (right) showing an area of hypoperfusion in the posterior temporal and inferior parietal cortex in an acute stroke patient who showed a pattern of performance in spelling similar to Case 5, attributable to partially impaired access to orthographic word forms and partially impaired access to orthography-to-phonology conversion mechanisms.

fore, some lexical representations that are both semantically and phonologically related to the target will be activated nearly as much as the target itself is activated. When the target lexical orthographic representation is unavailable due to damage at that level, the semantically and phonologically related representation might be activated for output instead [5].

Stroke participants sometimes show similar patterns of performance. For example, the patient whose scans are shown in Fig. 4 had fluent, grammatical but anomic speech, with disproportionately impaired reading and spelling, but intact auditory comprehension. Sentence repetition was impaired. This individual produced phonologically similar word errors in spelling (e.g. axe → axel) at the acute stage of stroke, as well as some phonologically implausible nonwords (e.g. church → chorch). Interestingly, this patient's area of ischemia, in the left inferior parietal lobule and superior temporal cortex is the same brain region where participants with lvPPA show the most focal atrophy. The previously described stroke patient RCM, whose frontal lesion spared the left temporo-parietal cortices also showed a similar pattern of performance in spelling after she improved in POC mechanisms after rehabilitation. This is consistent with the hypothesis that partially spared POC mechanisms can interact with partially spared lexical mechanisms for accessing word forms for output.

### 3.4. *Impairment at the level of the graphemic buffer*

In other cases equal impairment in spelling of words and pseudowords could be due to functional disruption at the level the graphemic buffer, a short-term memory system for holding a series of graphemes on-line while the word is spelled aloud or written. In such a case, we would expect all spelling tasks to be equally affected except direct copying of words or pseudowords [21]. Delayed copy transcoding entails showing the patient a word or pseudoword briefly, and then asking them to write the word from memory in the opposite case (upper vs. lower case). Even if they do not know how to spell the word, or do not have a lexical representation as in the case of pseudowords, they should normally be able to retain the sequence of graphemes long enough to spell the word. Participants with impairment at this level have trouble retaining the sequence of graphemes long enough to spell the word or pseudoword, even if they do accurately access the correct spelling (the lexical representation), both in oral and written spelling tasks. They make more errors per letter on longer words than shorter words, because longer words put more stress on the buffer. Because the buffer affects output after the lexical representation is accessed or the spelling is assembled, there are no significant effects of lexical parameters such as word frequency, concreteness, regularity, or grammatical word class (if the impairment is the only deficit in the spelling system), other than word length in graphemes. Errors are phonologically implausible nonword errors, consisting of insertions, deletions or transpositions of letters [21,22].

*Case 6* presented with such a profile. He is a 70-year-old man with college education first tested when he was 3 years post onset of nfvPPA. He was tested a second time a year later, by which time he was almost mute. There was no significant effect of lexicality (words vs. pseudowords (34.5% vs. 32.4%); orthographic probability (high vs. low: 93.3 vs. 81.3%), concreteness (concrete vs. abstract: 76.2 vs. 47.6%). More than 73% of his errors were phonologically implausible errors (PINs) the first time he was tested, and 79% of his errors were also PINs a year later on his second testing. These errors included deletions (e.g. priest → priet; caught → cauht), substitutions (e.g. decent → deciet), transpositions (e.g. nature → natuer), and mixed errors (e.g. fierce → furrage; above → aveu). He made similar errors on pseudowords (e.g. donsept → dorseat; croid → crier).

Stroke participants who show this identical pattern of errors have been described in the literature [21–24]. These participants have tended to have focal lesions involving the posterior parietal cortex [25] or visual association cortex [26]. However, in a recent study of 331 participants with acute left hemisphere ischemic stroke evaluated with various spelling tests and magnet-

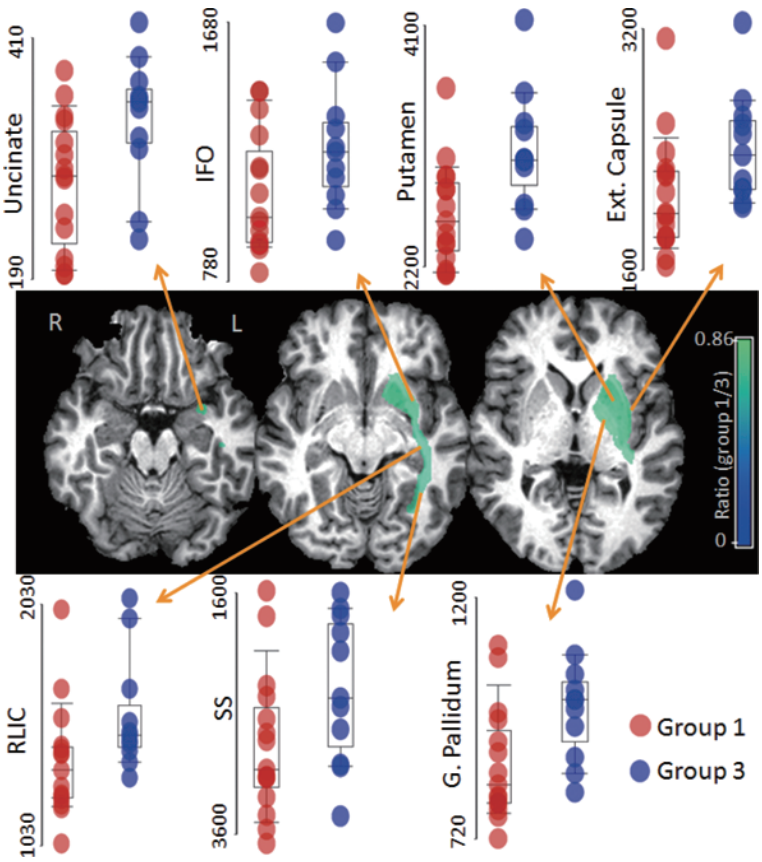

Fig. 5. Comparison between participants who made phonologically plausible errors (PPEs, group 1) and those who made a mixture of PPEs and implausible errors (group 3). Colors overlaid to T1-WI represent the ratio (group 1/3) of the volumes of the areas that showed statistical significant differences between groups ($p < 0.01$). Participants who made PPEs had greater atrophy of uncinate, inferior frontal occiptal fasciculus (IFO), saggital stratum (SS, that includes geniculostriate pathway and inferior longitudinal fasciculus), globus pallidum (G Pallidum), putamen, retrolenticular portion of internal capsule (RLIC) and external capsule, all in the left hemisphere.

ic resonance diffusion and perfusion-weighted imaging a voxel-wise statistical map showed that ischemia in posterior and inferior frontal and parietal cortices, subcortical white matter underlying left prefrontal cortex, lateral occipital gyrus, and caudate was associated with spelling impairments at the level of the graphemic buffer [27]. Functional imaging studies also provide evidence for an extensive network of occipital, parietal, and inferior frontal regions supporting a “visual-spatial sketch pad” as might be needed for any sort of buffer system for holding information that has inherently spatial extent [28–32]. Data from stroke participants who make errors that increase as a function of the number of letters from the center of the word toward the contralesional side, irrespective of the task (spelling, reading, backward spelling, mirror-reversed reading) provide evidence that information at the level of the graphemic buffer does have spatial extent [33].

### 3.5. Group imaging data

Participants were divided into 4 groups. Group 1 (15 participants) included those who made predominantly PPEs, due to severe impairment at the level of semantics and/or the orthographic word form, and relied on phonology-to-orthography conversion mechanisms for spelling. Cases 1–3 described in the previous sections fell into this group. Group 2 (of which there was only 1 participant, Case 4 outlined above) made mostly semantic errors, and showed the pattern sometimes referred to as “deep dysgraphia”. Group 3 (11 participants) made a mixture of PPEs and implausible errors, with frequent phonologically similar word errors, due to partially impaired access to orthographic word forms and partially impaired phonology to orthography conversion mechanisms, exemplified by Case 5 above. Finally, Group 4 (3 participants) showed impairment at the level of the graphemic buffer, exemplified by Case 6 above.

Figure 5 shows comparison for the two largest groups, 1 and 3. There were statistically significant volumetric differences in 7 brain regions, all in the left hemisphere. These included uncinate, inferior fronto-occipital fasciculus, and sagittal stratum (including geniculostriate pathway and inferior longitudinal fasciculus). All 7 regions were significantly more atrophied in group 1 vs. group 3. The results remained significant when regional volumes were normalized by the total brain volume.

## 4. Discussion

The above cases illustrate that the patterns of dysgraphia previously described after focal vascular lesions (stroke) can also be identified in neurodegenerative cases of PPA. Interestingly, there does not seem to be a one-to-one correspondence between the variant of PPA and the form of dysgraphia, but there are some correspondences between the location of brain atrophy associated with a particular variant of PPA and the affected component of the spelling system, that are consistent with the post-stroke dysgraphia literature.

For example, the combination of severely impaired POC mechanisms and impaired access to orthographic word forms (especially for verbs), with semantic errors in spelling despite good word comprehension was observed only in a nfvPPA patient, who had focal atrophy in posterior left inferior frontal cortex (BA 44/45). This same pattern of dysgraphia has been reported in detailed case studies of stroke participants with focal strokes in BA 44/45 [5,20]. Milder impairments in POC were observed in combination with impaired access to access to orthographic word forms in lvPPA participants, who have atrophy in left inferior parietal cortex. These participants made mostly phonological similar word errors. Similar patterns of dysgraphia have been observed in stroke participants with ischemia in left inferior parietal cortex.

Interestingly, the only PPA participants with impairment at the level of the graphemic buffer had nfvPPA. nfvPPA classically affects the posterior inferior frontal cortex, and this region was identified as critical for the graphemic buffer in a recent study of 331 participants with acute left hemisphere ischemic stroke evaluated with various spelling tests and magnetic resonance diffusion-weighted imaging and perfusion-weighted imaging [27]. That study revealed ischemic damage to many regions including posterior and inferior frontal and parietal cortex, subcortical white matter underlying prefrontal cortex, lateral occipital gyrus, and caudate were associated with acquired spelling impairments at the level of the graphemic buffer. All these regions have been associated with working memory functions (bilaterally), in functional neuroimaging studies.

Most notably, we found that left hemisphere brain regions where atrophy correlated with impairments in access to orthographic word forms and semantics (with reliance on phonology-to-orthography to produce phonologically plausible spellings of words) included the uncinate, inferior fronto-occipital fasciculus, sagittal stratum (including geniculostriate pathway and inferior longitudinal fasciculus), and other deep grey and white matter structures. These tracts have connections with the anterior and inferior temporal lobe and peri-rhinal cortex – areas of cortex known to be affected in svPPA [7]. Furthermore, these same white matter tracts have been found to be disproportionately atrophied in svPPA compared to other variants of PPA. Indeed, most of the participants with this pattern of dysgraphia (9/15) were classified as having svPPA. Others were not classifiable, on the basis that they did not meet criteria for any of the variants, but had only dysgraphia and anomia. One hypothesis is that this pattern of performance may turn out to be an early sign of svPPA. Surface dyslexia is one of the supporting features of svPPA [7]; "surface dysgraphia" may also turn out to be an important early diagnostic symptom.

Most current concepts of the neural networks underlying language include critical nodes for object semantics within the temporal cortex (often including a semantic hub in the anterior temporal lobe – the temporal pole, anterior portion of the superior and middle temporal gyrus and fusiform gyrus anterior to Brodmann's area 37) [34,38–45], and often a more distributed posterior frontal, middle temporal and inferior parietal network underlying action semantics [46]. In previous studies we proposed that access to orthographic word forms for output often depends on an interaction between (or summation of) partial information from semantics and partial information from POC mechanisms [17,18]. Connections between these semantic nodes in temporal cortex and areas critical for access to orthographic word forms (including posterior frontal cortex) and POC mechanisms (such as posterior frontal cortex, angular gyrus, and perhaps supramarginal gyrus) could be essential for such interactions. Disruption of these white matter tracts would plausibly result in reliance on a single component (e.g. POC mechanisms), depending on which component is preserved. In svPPA, POC mechanisms might be preserved because (as previous evidence indicates) POC mechanisms depend on posterior frontal cortex and inferior parietal cortex – regions that are relatively preserved in svPPA.

Considering the computational demands of spelling to dictation participants who make phonologically plausible errors on this task may do so because 1) the distributed components of the orthographic word form are *not accessible* together, or 2) the word form is not being activated appropriately from the impaired semantic representation (or from the spoken input), or 3) the orthographic word form (the "representation") is degraded in some way. Although it would be empirically difficult to distinguish these causes of PPEs, one can imagine that disruption of white matter tracts, leading to impaired connections between critical nodes of a network, could lead to any of these 3 causes of such errors.

Although there was not a one-to-one correspondence between the dysgraphia type and the PPA variant, at a group level the majority of participants with Pattern 1 (reliance on POC mechanisms) had svPPA, and the majority of participants with Pattern 3 (impaired POC mechanisms as well as partially impaired access to orthographic word forms) had lvPPA. The majority of participants with impairment at the level of the graphemic buffer (Type 4) had nfvPPA. The only patient with abolished POC mechanisms and production of predominantly semantic errors in writing (Type 2) also had nfvPPA. These results indicate that the dysgraphia subtype might provide a diagnostic clue early in the course of PPA. For example, Case 1 did not initially meet criteria for svPPA as he had intact word and object comprehension, but had predominantly PPEs in spelling. Later he developed severe word and object comprehension deficits with clear svPPA. Likewise, Case 2 initially did not meet criteria for svPPA, because of intact word and object comprehension. At that time, she did show the Type 1 pattern of performance, with predominantly PPE's. Later, when she maintained this pattern of performance in spelling, albeit with lower spelling accuracy, she was diagnosed with svPPA.

Identification of the cognitive and neural substrates that are impaired in cases of dysgraphia in PPA can not only provide a better understanding of the neural substrates of spelling, but may also provide clues to more effective treatment approaches. Although PPA is a progressive condition, intervention can be helpful in at least temporarily improving production of trained sets of words in both speech [47] and writing [48], which in turn may improve the individual's quality of life, sense of accomplishment and perhaps contribute to their maintenance of language functions over time. Other reasonable goals of treatment might be to reduce the rate of language decline and to provide effective alternative communication strategies.

## Acknowledgments

The research reported in this paper was supported by NIH (NIDCD), through RO1 DC 05375 and RO1 DC 03681 (AEH); UL1 RR 025005 from NCRR/NIH and NIH Roadmap for Medical Research (AVF), and UK MRC Clinical Scientist Fellowship G0701888 (JC). We gratefully acknowledge this support and the involvement of the participants.
